# Prevention of genital herpes in a guinea pig model using a glycoprotein D-specific single chain antibody as a microbicide

**DOI:** 10.1186/1743-422X-1-11

**Published:** 2004-11-23

**Authors:** Jianmin Chen, Sanat K Davé, Anthony Simmons

**Affiliations:** 1 University of Texas Medical Branch, Galveston, Texas, USA

## Abstract

**Background:**

Genital herpes (GH) is a recurrent sexually transmitted infection (STI) that causes significant morbidity and is also the major source of herpes simplex virus (HSV) in cases of neonatal herpes. Vaccination is a current goal which has had limited success so far in preventing GH and microbicides offer an attractive alternative. Treatment of primary disease cannot prevent establishment of latent infections and thus, cannot prevent subsequent recurrent disease. Recently, many of the molecular events leading to entry of HSV into cells have been elucidated, resulting in the description of a number of herpesvirus entry mediators (HVEMs) that interact with HSV glycoprotein D (gD) on the surface of virions. Described here is a strategy for interrupting the spread of HSV based on interfering with these interactions. The hypothesis addressed in the current report was that single chain antibody variable fragments (scFv) that interrupt associations between gD and HVEMs would not only prevent infection in vitro but could also be used as microbicides to interfere with acquisition GH.

**Results and Conclusions:**

Here we show that a scFv derived from a particular hybridoma, DL11, not only inhibits infection in vitro but also prevents development of GH in a guinea pig model when applied intravaginally in an inert vehicle. Comparison of different anti-gD single chain antibodies supported the hypothesis that the activity of DL11-scFv is based on its ability to disrupt the associations between gD and the two major receptors for HSV, nectin-1 and HveA. Further, the results predict that bacterial expression of active single chain antibodies can be optimized to manufacture inexpensively a useful microbicidal product active against HSV.

## Background

GH is generally caused by HSV type 2 (HSV-2), though HSV type 1 (HSV-1) is increasingly recognized as a significant cause of primary infections [1]. Throughout the last few decades there were substantial advances in understanding the epidemiology of genital HSV infections. Primary infection is almost always self-limited but on healing virus is not eliminated from the host but rather, viral genomes remain in a latent (dormant) state in sensory neurons innervating initially infected skin and mucous membranes [2,3]. The significance of latency is that it is a reservoir of infection that can periodically reactivate, causing virus to travel down nerve fibers to skin or mucous membranes in the dermatome of primary infection. This may be manifest clinically as recurrent GH or more frequently, causes unrecognized shedding of infectious HSV [4-7] which despite being unrecognized is responsible for the majority of new HSV-2 infections [8]. The epidemiology is further complicated by the fact that many primary infections are asymptomatic or unrecognized, which has the important implication that the first clinical presentation of GH, often referred to as the initial episode, may be caused by a recurrence of a prior asymptomatic primary infection [9].

In the latter half of the 20^th ^century, there were great strides in antiviral therapy for GH but unfortunately, treating primary disease does not prevent establishment of infection [10] and thus, cannot prevent subsequent recurrent disease. Barrier contraception provides some protection but its efficacy remains unclear [11] owing to the complex nature of HSV pathogenesis, in which virus is shed frequently and asymptomatically from multiple sites below the waist [5]. Hence condoms are not as effective at preventing transmission of GH as they are for other sexually transmitted infections. Vaccination is a current goal which has had limited success to date. A recent trial of a glycoprotein D-based sub-unit vaccine protected only double (HSV-1 and 2) seronegative women but not men [12]. Further, protection was mainly measured by prevention of primary disease rather than infection. It is known that treating primary disease does not prevent establishment of latency and consequently, the long term efficacy of this vaccine against subsequent recurrences remains unknown.

Thus, the number of strategies for preventing sexual transmission of GH is limited. Recently, there has been considerable interest in topical microbicides as a potentially attractive alternative to vaccination for prevention of sexually transmitted infections, including GH [13]. Women are able to control the use of vaginal microbicides and several products are currently being used or tested, including acid buffers and sulphated polymer-based inhibitors or surfactants [14] like nonoxynol-9 (N-9) [13]. N-9 has been used as a spermicide for 30 years and was thought to provide some protection against gonorrhea and chlamydia, a view was recently proven to be erroneous [14]. A major factor limiting the efficacy and long-term viability of N-9 and similar chemical compounds as topical agents is their irritant effects on the vaginal epithelium [15]. Further, recent data suggest that N-9, contrary to prior belief, is not effective at protecting against HIV but rather it was shown to increase rather than decrease the risk of acquiring HIV in some populations studied, particularly women at high risk of infection [14].

**Figure 1:**
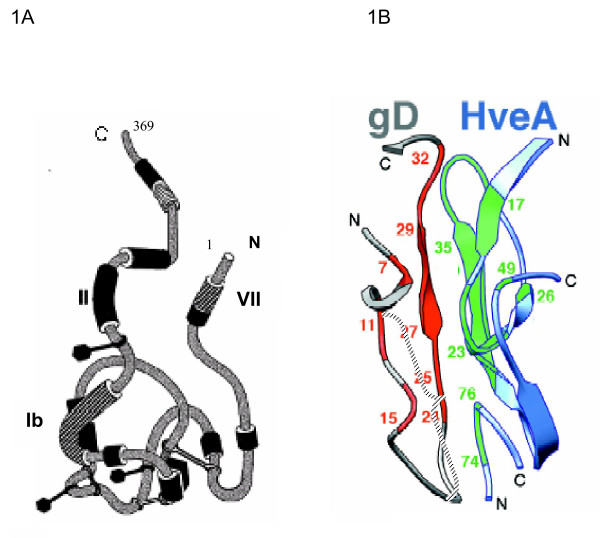
Panel A: Hypothetical model illustrating the antigenic structure of gD and demonstrating the clustering of antigenic sites into seven groups, as defined by locations of amino acids bound by various monoclonal antibodies. Disulphide bonds location defined by braces. Diagram adapted with permission from Nicola et al, 1998 [22]. Of particular relevance to this study are the locations of sites VII (amino acid residues 11–19), which is bound by antibody 1D3, and site Ib, a discontinuous epitope that includes residues 222 to 252 that is bound by antibody DL11. Panel B: Diagram showing interface between N-terminal amino acids of gD and HveA and the approximate residues (blue) bound by monoclonal antibody 1D3 and, by inference, 1D3 scFv (adapted with permission from Connolly et al, 2003 [19].

An evolving strategy that may be useful for preventing specific sexually transmitted viral infections is blocking of virus entry into cells or subsequent inhibition cell-to-cell spread. Many of the molecular events leading to entry of HSV into cells have now been unraveled, resulting in the description of two prominent cell-surface herpesvirus entry mediators (Hve-A and nectin-1, also known as Hve-C) that interact with HSV glycoprotein D (gD) on the surface of virions [16-20]. In a recent study [21], nectin-1 was shown to be expressed in the vaginal epithelium of humans throughout the estrous cycle. In contrast, in mice nectin-1 was expressed in vaginal epithelium only during the stage of estrous at which they are susceptible to HSV. Using a mouse model of GH, pre-incubation of HSV-2 with soluble recombinant nectin-1 was shown to block entry of virus through vaginal mucosa [21], suggesting the importance of nectin-1 in mediating entry of HSV into the female genital tract. Hve-A and nectin bind to conformationally overlapping regions of gD and we were able exploit this information together with the results of prior studies that had mapped the sites on gD recognized by a panel of monoclonal antibodies [22-26]. Antibody DL11 was of particular interest because it binds to an epitope on gD that blocks the interactions between gD and both Hve-A and nectin-1 [19] (figure 1). We show here that a single chain antibody variable fragment (scFv) constructed from DL11 neutralizes HSV infection in vitro, inhibits cell-to-cell spread of virus and can be used to prevent infection in a guinea pig model of GH.

## Results

### Construction and expression of single chain antibodies against gD

**Figure 2:**
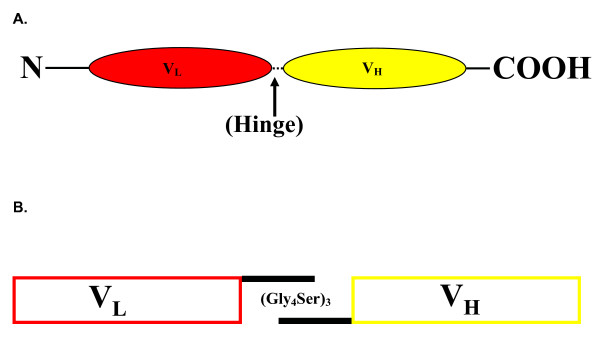
Panel **A: **Structure of an scFv cassette spliced using a (Gly_4_Ser)_3 _hinge. Panel B. Alternative glycine codons were used in the overlapping region of the hinge to avoid production of completely overlapping regions, thereby generating a sub-optimal (Gly_4_Ser)_2 _hinge.

**Figure 3:**
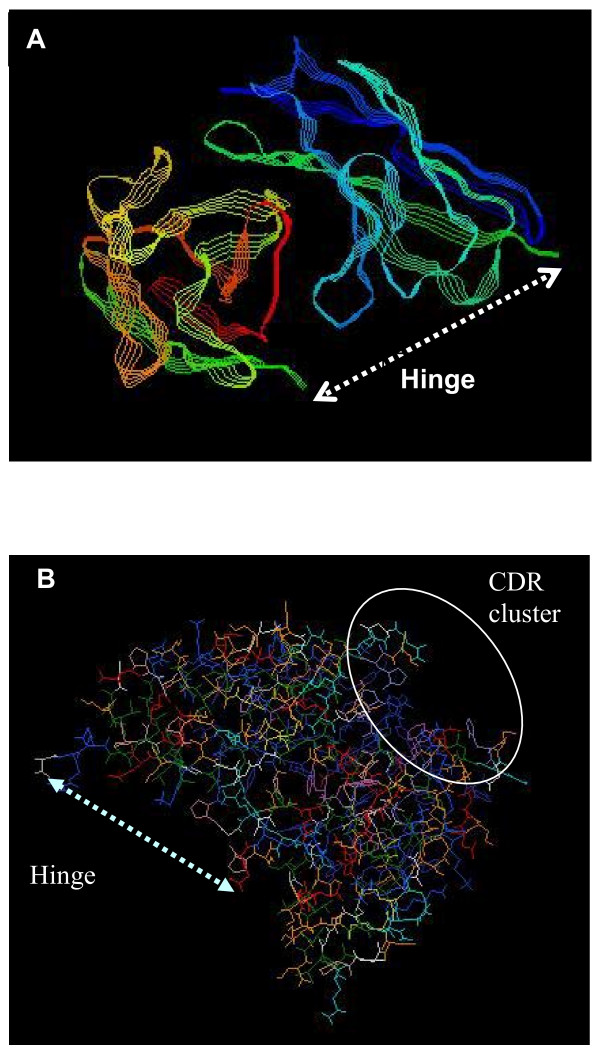
Single plain view of a 3-D model of DL11 scFv, showing its predicted structure. **Panel A: **Strand view, colored by group, demonstrates relative orientation of the kappa (top) and gamma (bottom) chains, which shows the positions of residues to which the (Gly_4_Ser)_3 _hinge is attached. **Panel B: **Wireframe image illustrating hinge attachment sites on one side of the molecule (linked by dashed line) and clustering on the opposite side (inside the circle) of the complementary determining regions (CDRs) predicted by the Kabat antibody database. The clustering of CDRs suggests correct conformation of the molecule with formation of an antigen binding site.

**Figure 4:**
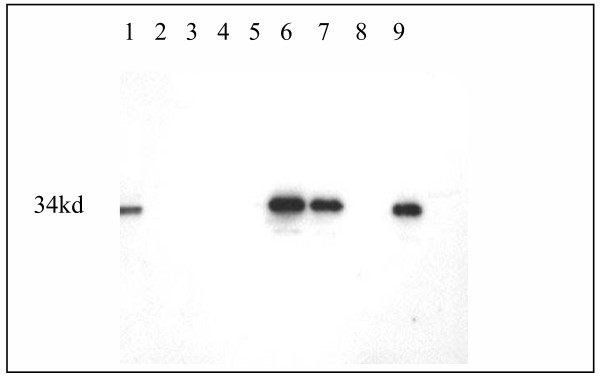
Western blot demonstrating expression of DL11 scFv by E, Coli. BL21 cells were transfected with p-TOPO10 containing the scFv cassette. Bacterial lysates were purified using a nickel chelation column and the reaction with anti-V5 of total lysates and various fractions from the column are shown. Lane 1, unpurified total bacterial lysate; lane 2, nickel column flow through; lanes 3 and 4, saline washes; lane 6, eluate from Ni beads; lane 7, bacterial supernatant; lane 8, scFv remaining on nickel column after elution; lane 9: supernatant from un-induced bacteria.

Four from the panel of anti-HSV gD hybridomas available were selected for scFv construction based on the known locations of their epitopes [22] (summarized in figure 1) and knowledge about the neutralization properties of the antibodies produced by them. Of particular note are the properties of DL11, which neutralizes both HSV-1 and HSV-2 in the absence of complement and antibody binding to its conformational epitope is known to disrupt the interactions of gD both with Hve-A and nectin-1. Also 1D3 binds to a group VII neutralizing epitope that directly interferes with the interface between gD and HveA (figure 1B). A fifth scFv cassette, against carcinoembryonic antigen (CEA) was excised from a plasmid encoding an anti-tumor chimeric T-cell receptor, kindly provided by Hinrich Abken (Cologne University, Germany). For production of cDNAs, individual V_L _and V_H _regions from each hybridoma were reverse transcribed using primers near the V_H_-C_H _and V_L_-C_L _junctions. For PCR cloning these primers were paired with a panel of degenerate primers derived from V_H _or V_L _signal sequences (Table 1) that were able to amplify all hybridoma heavy and light chains tested so far (14/14) irrespective of antibody class or subclass (data not shown). PCR products were sequenced directly to facilitate design of new primer sets allowing, on re-amplification of hybridoma cDNAs, elimination of degenerate primer sequences introduced in the first reaction and introduction of 2/3 of a 15 amino acid hinge region comprising three repeats of four glycine and one serine residues (Figure 2). V_L _and V_H _are not covalently linked in nature but flexible hinges of this design and length were shown previously [27] to allow reconstruction of antibody binding sites when V_L _and V_H _are linked end-to-end (figures 3, 4). Finally, the PCR products containing the overlapping hinge regions were ligated, PCR amplified and the resultant scFv cassette was TA cloned into pCR2.1TOPO. To generate the desired single chain antibodies, the cassettes were subcloned into the bacterial expression vector pET101-D. An antibody modeling algorithm, verified by the locations of the complementary determining regions, was used to predict the 3-D structures of all four of the anti-gD single chain antibodies. The results were consistent with reconstitution of the original antigen binding sites (e.g. figure 3, DL11; others not shown).

**Table 1:** Degenerate PCR primers used for amplification of V_L _(kappa) and V_H _(gamma).

**Nomenclature**	**Primer sequences used for PCR reactions**
*Signal sequence/framework primers*	
Kappa 1	GGTGATATCGTGATRACMCARGATGAACTCTC
Kappa 2	GGTGATATCWTGMTGACCCAAWCTCCACTCTC
Kappa 3	GGTGATATCGTKCTCACYCARTCTCCAGCAAT
Kappa 4	CTGWTGTTCTGGATTCCTG
Kappa 5	GTGCTCTGGATTCGGGAA
Kappa 6	TCAGCTTCYTGCTAATCAGTG
Kappa 7	TGGGTATCTGGTRCSTGTG
Kappa 8	GTTTCMAGGTRCCAGATGT
Kappa 9	TGTTTTCAAGGTRCCAGATGT
Kappa 10	CTSTGGTTGTCTGGTGTTGA
Kappa 11	TGCTKCKCTGGGTTCCAG
*C region kappa primer*	TGGTGGGAAGATGGA
*Signal sequence/framework primers*	
Gamma 1	GAGGTGAAGCTGCAGGAGTCAGGACCTAGCCTGGTG
Gamma 2	AGGTVMAACTGCAGVAGTCWGG
Gamma 3	AGGTVVAGCTGCAGVAGTCWGG
Gamma 4	ACTGCAGGTRTCCACTCC
Gamma 5	RCTACAGGTGTCCACTCC
Gamma 6	GCYACAGMTGTCCACTCC
Gamma 7	ACTGCAGGTGTCCTCTCT
Gamma 8	RCTRCAGGYGTCCACTCT
Gamma 9	CCAAGCTGTGTCCTRTCC
Gamma 10	TGTTGACAGYCVTT CCKGGT
Gamma 11	TAYTTTAAAARGTGTCMAGTGT
Gamma 12	CTYTTAAAAGGKGTCCAGWG
Gamma 13	CYTTTAMATGGTATCCAGTGT
Gamma 14	ATGGCAGCWGCYCAAAG
Gamma 15	CTTTTAAAAGWTGTCCAGKGT
Gamma 16	CTTCCTGATGGCAGTGGTT
C region gamma primer	TAACCCTTGACCAGGCATCC

Key to degenerate nucleotides: R = A+G; M = A+C; W = A+T; K = G+T; S = G+C; Y = C+T; H = A+T+C; B = G+T+C; D = G+A+T; N = A+C+G+T; V = G+A+C

### Bacterial expression and extraction of anti-gD single chain antibodies

**Figure 5:**
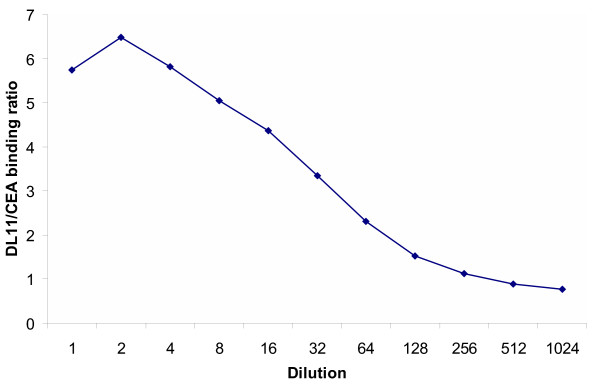
Binding of scFv to plastic bound gD. Binding ratios of DL11 scFv to gD compared with an irrelevant (CEA) scFv at the same protein concentrations.

The single chain antibodies were expressed in E. Coli strain BL21 using pET101-D (Invitrogen), which attaches hexa-His and V5 tags to expressed proteins for their isolation and identification. Bacteria were induced with IPTG, centrifuged and the supernatants tested for the presence of scFvs by western blotting using anti-His antibody (figure 4). Bacterial pellets were sonicated in phosphate buffered saline to release inclusion bodies and proteins were solubulized by addition of 6 M guanidine (BL21). Nickel bead chelation was used to extract the His-tagged protein. Western blots of eluates from nickel beads (e.g. DL11 scFv from DL21; Fig. 4, lanes 6 and 7) identified scFvs that were released by this procedure. They were generally isolated at concentrations of 500–1000 µg/ml from BL21. Re-folding and intra-chain disulphide bond formation were maximized by gradually reducing guanidine concentration by step-wise dialysis from 6 M initially to 3 M, then 2 M, 1 M, 0.5 M and finally 0 M, with addition of L-arginine and oxidized glutathione (GSSG) in final two steps [28]. The ability of the single chain antibodies produced in this way to bind their target antigen was tested by determining their reaction with plastic bound gD by ELISA. Binding ratios were calculated in relation to the background binding of CEA scFv (e.g. DL11-based scFv; figure 5)

### Selected anti-gD single chain antibodies neutralize HSV in vitro

**Figure 6:**
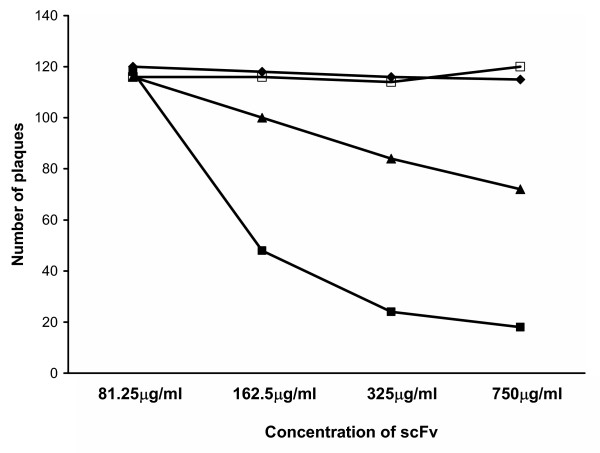
Specific reduction of HSV-2 plaque numbers by incubation of virus with anti-gD scFv. Vero cells were pre-incubated with approximately 120 PFU HSV-2, strain G with single chain antibodies generated from hybridomas D11 (¦), 1D3 (?), DL2(?) and an irrelevant CEA-specific construct (?).

**Figure 7:**
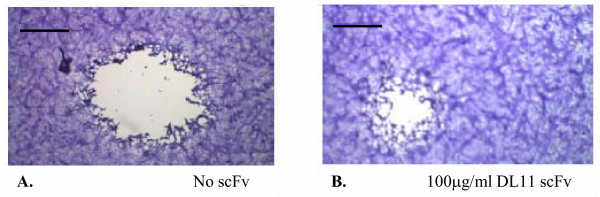
Reduction in plaque size in the presence of DL11 scFv. Mean plaque size in absence of scFv (Panel A) was 1.9 ± 0.4 mm compared with 0.95 ± 0.3 mm in presence of 100 mg/ml DL11 scFv (Panel B). Figures represent mean of 100 plaques ± standard deviation. Bar = 1 mm.

The hypothesis that selected single chain antibodies can block infection of cells *in vitro *by reacting with an epitope that disrupts the interface between gD and HVEMs was tested by comparing the activities of the various bacterially expressed anti-gD scFv in a Vero cell-based HSV-1 plaque reduction assay. A scFv directed against an epitope on carcinoembryonic antigen was included as an unrelated control. The results showed that pre-incubation of virus with DL11 and 1D3 scFvs inhibited plaque formation with decreasing efficiency. DL6 scFv showed minimal but reproducible activity (data not shown), whereas the other scFvs tested (DL2 and CEA) had no plaque reducing capability at all (figure 6). Against HSV-2, only DL11 showed neutralizing activity in a similar plaque reduction assay (data not shown), confirming the type common nature of its epitope. In addition to inhibition of plaque formation, pre-incubating HSV-2 with 100 µg/ml DL11 caused an 80% reduction in plaque numbers and a ~50% reduction (figure 7) in the size of plaques (0.95 ± 0.3 mm with DL11scFv vs. 1.9 ± 0.4 mm without, respectively). The same was true for HSV-1 and DL11 (not shown). It was concluded that DL11scFv could not only block infection of cells with HSV but also was able to inhibit cell-to-cell spread of virus.

### Protection against HSV type 1 and type 2 GH by administration of a DL11-based single chain antibody before infection with virus

The HSV type-common and startling in vitro activities of single chain antibodies derived from hybridoma DL11 prompted us to examine the ability of DL11scFv to protect against vaginal HSV disease, using a well established guinea pig model of GH [29,30]. The vehicle selected for these preliminary studies was 1% carboxymethylcellulose because this is an inert compound that is used for its viscosity in our routine plaque assays.

**Figure 8:**
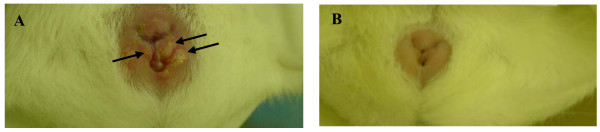
Effect of DL11 scFv on HSV-1 genital disease in guinea pigs. Panel A: Blisters of GH 5 days after instillation of HSV-1 into vaginal vault. Several areas of ulceration with surrounding erythema are visible bilaterally (e.g. arrows); Panel B: Complete protection against HSV-1 by prior instillation, immediately before HSV challenge, of 1 ml CMC containing DL11 scFv (500 µg/ml).

A pilot experiment was done with HSV-1, in which BL21 produced DL11 and DL2 single chain antibodies (0.5 mg/ml) were each instilled into the vaginas of guinea pigs (1 ml/animal). Approximately 20 seconds later the guinea pigs were challenged with 5 × 10^6 ^PFU HSV-1, strain SC16 and monitored for development and severity of primary disease. The result (figure 8) showed that DL11-based scFv completely protected the animal from lesion development whereas DL2-based scFv appeared to have, as expected, no effect.

Next a more ambitious test of microbicidal activity was attempted, using HSV-2 rather than HSV-1 and a longer interval (20 minutes) between scFv instillation and challenge (Table 2). Two groups of 20 guinea pigs were each administered either DL11 or DL2 (control) scFv (1 ml/guinea pig). All animals were challenged with 10^6 ^PFU of HSV-2, strain G and monitored daily as before. All except one animal were completely protected by DL11 scFv compared with DL2 scFv, all of which developed moderate to severe disease, scored as described in methods (p = <0.0001; Mann Whitney test).

**Table 2:** Prevention of GH in guinea pigs by DL11 scFv.

**Pre-treatment**	**Severity of lesions**	**Mean lesion score (n = 20)**
500 µg/ml DL2 scFv 20 minutes prior to infection	2, 2, 3, 3, 3, 3, 3, 4, 4, 4, 4, 4, 4, 4, 4, 4, 4, 4, 4, 4.	3.55 ± 0.153 *
500 µg/ml DL11 scFv 20 minutes prior to infection	0, 0, 0, 0, 0, 0, 0, 0, 0, 0, 0, 0, 0, 0, 0, 0. 0, 0, 0, 3.	0.15 ± 0.15 *

* p < 0.0001 (Mann-Whitney test)

## Discussion

In practice, the rapidity of isolation and cloning of scFv into a bacterial expression vector (approximately one week) by the procedure described here allowed expeditious activity assessments to be made for the different constructs. Bacterial protein expression systems are widely used for the production of recombinant proteins and problems are often encountered with disulphide bond formation. Whilst there are no covalent bonds between heavy and light chain sequences in immunoglobulin hypervariable domains, intra-chain disulphide bonds can, to varying degrees among different antibodies, influence conformation of the antigen binding site [31]. Thus, failure to form intra-chain disulphide bonds has the potential to disrupt antigen binding and could also detrimentally affect stability of the molecule. For this reason, a previously reported method [28] was adapted to promote formation of intra-chain disulphide bonds in vitro, using protein extracted from bacterial inclusion bodies. After isolation of inclusion bodies by sonication of bacteria, proteins were solubulized with 6 M guanidine hydrochloride, the concentration of which was gradually reduced to zero by a stepwise daily dialysis routine. In all cases we tried, this procedure generated soluble hexa-His tagged single chain antibody fragments, which have an approximate molecular weight of 34 kD, at concentrations of approximately 750 – 1000 µg/ml. Precipitation of proteins in the final dialysis step tended to occur above concentrations of 1000 µg/ml and was prevented by careful monitoring of the sample volume to ensure concentrations stayed below this critical threshold.

Entry of HSV into cells is known to be mediated through interactions between gD and 3-O-sulfated heparan and one or more specific entry mediators, HveA, nectin-1 and nectin-2 [32]. Overall, the results of plaque reduction assays in vitro were compatible with the hypothesis that significant interference with the binding of gD with HVEMs can be achieved with a single chain variable fragment selected according to the known properties of their parent antibodies. This is the first direct evidence that neutralization of HSV can be a property of certain antigen binding domains alone, a corollary of which is that no other regions of the antibody need be required to neutralize the virus. Of the five scFvs tested in this study, the most effective was produced from antibody DL11, which is known to interfere with binding both to HveA and nectin-1. Neutralization in vitro by a scFv constructed from antibody 1D3, which binds to a site on gD that overlaps the binding site for HveA, was significantly less efficient than that seen with DL11 scFv, presumably because, in the presence of 1D3 scFv, HSV was still able to utilize nectin-1 as a receptor. As expected, scFvs derived from DL2, a non-neutralizing monoclonal antibody, and a scFv reactive against CEA showed no activity in the plaque reduction assay. The weak activity of the construct made from DL6 correlates with the known weak neutralizing property of the native antibody, which is presumed to be the result of conformational changes induced it's by its binding to gD.

The data presented here correlate with the prior finding that mice can be protected against HSV-2 by topical administration of antibody [33,34] and a subsequent report from Zeitlin et al [35] that mice were protected against HSV-2 transmission by intravaginal administration of an IgG_2a _monoclonal anti-gD antibody and its IgA switch variant. Here, these observations are extended in several respects. The epitope on gD recognized by the most effective scFv, that constructed from antibody DL11, was defined as one that interferes with binding of gD with two major mediators of herpesvirus entry into cells, namely HveA and nectin-1. In this respect, attention is drawn to the recent report of Linehan et al [21] that nectin-1 is expressed in the genital tracts of mice and humans and soluble nectin-1 can block entry of HSV into vaginal epithelium. These data [21] together with the unprecedented protection of guinea pigs by DL11scFV shown here strongly implicate nectin-1 as a critical mediator of HSV the entry genital epithelium and in fact it is suggested here that nectin-1, rather than other herpesvirus entry mediators, likely play a dominant role in genital tract infection. The protective activity of a scFv established with certainty that the constant regions of anti-gD antibody molecules are not required for protection against HSV. This finding has the important consequence of eliminating the complement binding activity of IgG, which will greatly limit the potential for unwanted inflammatory side effects of topically administered anti-gD preparations, an important advantage if they are to be used clinically. The specific nature of anti-herpes scFv and the ability to choose an inert formulation has two potential advantages over other microbicides. First is selected high specific activity against HSV and second is that they are not irritating to the genital tract. Their murine derivation is not anticipated to be a problem with topical use, but humanization of the hypervariable region is possible by grafting the complementary determining regions onto a human framework, This is an option should their systemic use ever be considered. Of particular interest may be the use of microbicidal gels prior to delivery for the prevention of neonatal herpes. The inert nature of single chain antibodies, combined with a suitable vector, should enable their widespread use in this context among HSV-2 seropositive mothers. These are important considerations given the high prevalence of GH and its frequent asymptomatic nature.

In summary, we believe that single chain antibodies against HSV merit further study and development as topical microbicides. The production of active molecules in bacteria makes their use a feasible and relatively inexpensive prospect.

## Conclusions

Single chain antibodies against HSV gD could be synthesized readily from several IgG secreting hybridomas using degenerate immunoglobulin heavy and light chain immunoglobulin primers that hybridized to regions flanking the complementary determining regions, which determine antigen specificity.

Two mechanisms of interference with infection were evident when DL11 scFv was examined in detail. First, the number of plaques produced by virus could be inhibited by up to 90% when reacted with HSV prior to infection of Vero cells, indicating that scFv neutralized virus prior to establishment of productive infection. This result also suggested that nectin-1 and HveA, the binding of which are both blocked by DL11, are the main mediators of virus entry into Vero cells. 1D3, which interferes specifically with the interface of gD with HveA, was effective to a lesser extent. Second, in addition the striking ability of DL11 scFv to neutralize virus inoculums, this particular construct reduced plaque size significantly, from which it was concluded that cell-cell spread of HSV was also inhibited. This observation could have implications for therapeutic use of single chain antibodies in the future and may have enhanced the performance of DL11 scFv as a microbicide in the guinea pig model. This result was mediated by suboptimal scFv concentrations for virus neutralization, implying that lower concentrations of DL11 scFv may be required to interfere with intercellular spread of virus than to block entry.

The finding that DL11 scFv was active for 20 minutes, the maximum time tested, when instilled into the vaginal vault was considered encouraging for future development of scFv as microbicides and the observation merits further consideration of the vehicle used. Slow release formulations may be appropriate depending on their cost. Overall, it appears that selected single chain antibodies are promising candidates for interfering with binding of gD to HVEMs and studies in a guinea pig model of GH suggest that they may comprise a plausible strategy for preventing transmission of GH.

## Methods

### Generation of scFvs

Single chain antibodies were constructed from four anti-gD secreting hybridomas, DL11, DL6, DL2 and 1D3. An additional scFv, directed against carcinoembryonic antigen (CEA) served as an independent control. Messenger RNAs from 5 × 10^5 ^- 10^6 ^hybridoma cells were isolated using Trizol (Invitrogen, CA) and cDNAs were generated by reverse transcription with Taq polymerase ('Expand High Fidelity Taq polymerase' ; Roche, IN). RT was primed with anti-sense oligonucleotides designed to anneal either to mouse kappa light chain or heavy chain constant region sequences, just downstream of the J-C junction (table 1). Light and heavy chain hypervariable regions (V_L _and V_H_) were amplified by priming 'sense' PCR reaction products with panels of oligonucleotides (OGNs) designed from Kabat database sequences to be complementary to kappa (light chain) and gamma (heavy chain) signal or framework sequences (table 1). In practice, pools of 11 degenerate OGN sequences were found to be sufficient to prime 100% of kappa chain reactions (14/14 hybridomas regardless of subclass). Similarly, a pool of 14 degenerate OGNs successfully amplified the gamma chains from these hybridomas. From each hybridoma, the resulting V_L _and V_H _cDNAs were sequenced and new specific primers were designed each of which included 2/3 of the fifteen amino acid (Gly_4_Ser)_3 _flexible hinge region, allowing the variable regions to be amplified and spliced together reconstituting the antigen binding site on reconformation (figures 2, 3). To prevent complete overlap of the complementary hinge sequences, which would result in the introduction of a sub-optimal 10 amino acid (Gly_4_Ser)_2_intervening segment, alternative glycine codons were used in each component of the hinge. Four of the scFvs were TA cloned into the bacterial expression vector pET101/D-TOPO (Invitrogen, Carlsbad, CA) which generates hexa-His tagged proteins after expression in vitro.

### Expression of single chain antibodies in bacteria

Proteins were expressed in IPTG-induced E. Coli BL21 [DE3] (Invitrogen), released by sonication in PBS and inclusion bodies were separated by centrifugation. Proteins in inclusion bodies were solubulized with 6 M guanidine HCl and purified by metal chelation. A stepwise dialysis procedure with addition of GSSG (oxidized glutathione; Sigma) and L-arginine in the final two steps was used to assist in the formation of intra-chain disulphide bonds in order to optimize re-conformation and stability of the scFvs [28]. Protein concentrations were measured using the BCA method (Pierce).

### ELISA to quantify binding of scFv to gD

Microtiter plate wells were coated with soluble gD (6 µg/ml) and then blocked with 1% skimmed milk. After incubation with serial two-fold dilutions of scFv, binding was detected with anti-V5, the alternative tag on the scFv, because the recombinant gD used in the assay was, like the single chain antibodies, tagged with hexa-His. Binding ratios were calculated in relation to an irrelevant (CEA-specific) scFv.

### Virus growth, titration and plaque neutralization assays

HSV-1 (strain SC16) and HSV-2 (strain G) were grown and titrated in Vero cells as described [36,37]. Titers were determined using a standard plaque assay [38]. Cells were grown and maintained in Dulbecco modified Eagle medium supplemented with 10% (growth medium; GM) or 1% (maintenance medium; MM) fetal bovine serum. A plaque reduction assay was done in Vero cells to assess the neutralizing capabilities of each scFv. Briefly, 100–200 plaque forming units (PFU), diluted in MM, of either HSV-1 (strain SC16) or HSV-2 (strain G) were incubated at room temperature for 1 hour with serial ten-fold dilutions of each scFv in a total volume of 1 ml. After gentle shaking with 3 × 10^6 ^Vero cells for a further 1 hour the samples were plated in 6 cm dishes (Nunc) in a total volume of 5 mls of GM containing 2% carboxymethylcellulose (CMC). Plaques were enumerated after 3 days incubation at 37°C in a 5% CO_2 _atmosphere.

### Guinea pig model of GH

The microbicidal properties of scFv were tested using a guinea pig model of GH. Female outbred Hartley guinea pigs weighing 350–400 grams were obtained from Charles River laboratories (Wilmington, MA). Prior to inoculation of each guinea pig with virus, the introitus was opened with a calcium alginate swab moistened in physiological saline and 1 ml of 1% CMC containing either DL2 scFv or DL11 scFv at a final concentration of 500 µg/ml, was instilled using a pipette with a plastic tip. CMC was used as a vehicle to facilitate retention of the scFv in the vaginal vault. At various times thereafter, animals were challenged with 10^6 ^PFU HSV-1 (strain SC16) or HSV-2 (strain G). Over the ensuing two weeks lesions were scored on a scale of 0–4 (0 = no lesion; 1 = erythema and swelling only; 2 = small vesicles <2 mm; 3 = coalescent or large vesicles >2 mm; 4 = ulceration and maceration). All experiments were done according to the guidelines laid down in The NIH Guide for Care and Use of Laboratory Animals and were approved by the Institutional Animal Care and Use Committee.

## Competing interests

The author(s) declare that they have no competing interests.

## Authors' contributions

AS conceived and coordinated the work described and wrote the manuscript. JC was responsible for the experiments described and SKD provided technical support.
